# A Suggestion for the Good of the Cause

**Published:** 1888-11

**Authors:** 


### EDITORIAL DEPARTMENT.

F. E. DANIEL, M. D., EDITOR AND PUBLISHER.

THIS JOURNAL, by unanimous vote of the members, was made the Official Organ of the Austin District Medical Society.

COLLABORATORS:

*Dr. R. M. Swearingen, Austin.*
*Prof. B. E. Hadra, M. D., Galveston.*
*Prof. Geo. Cupples, M. D., San Antonio.*
*Dr. T. C. Osborn, Cleburne, Texas.*
*Dr E. J. Doering, Chicago.*
*Dr. E. J. Beall, Fort Worth.*
*Dr Odo Betz, Germany.*
*Dr. J. W. McLaughlin, Austin.*
*Dr. T. J. Tyner, Austin.*
*Prof. J. F. Y. Paine, M. D., Galveston.*
*Dr. R. H. L. Bibb, Mexico.*
*Dr. T. J. Bennett. Austin.*
*Dr. Bat Smith, Wharton.*
*Dr. E. Meierhof, New York.*
*Prof. W. B. Rogers, M. D., Memphis, Tenn.*

### A SUGGESTION FOR THE GOOD OF THE CAUSE.

The comparatively slim attendance on the meetings of the Texas State Medical Assocation in the past three or four years, has been a matter of surprise and regret; and the very small percentage of the physicians of the State who have connected themselves with the organization, has been much commented upon. It is very discouraging, indeed, that after twenty years of labor on the part of a few zealous workers—during which time the Texas State Medical Association has attracted wide attention, and elicited much praise from the medical press for the quantity and quality of its work—and, notwithstanding the ephemeral enthusiasm in the cause engendered by spirited journalism, the result of which was to attract large additions to the roll of members, the Association numbers not over eight or ten per cent. of the estimated medical population of the State ! At Belton, in '84, Houston in '85, Dallas in '86, and Austin in '87, the addition of new members aggregated some two hundred and fifty, and yet, to-day, the roll shows only a membership of some four hundred and twenty.

Why is this? We have commented on this before, and sought to show that the reason of non-cohesion could be found in the fact that the Association will meet one year at one point, absorbing a large proportion of the material in the way of eligible physicians in that vicinity, and the next year, will meet at another point, so far off that only the most zealous of the new members last acquired will attend the meeting. For this reason, we have advocated meeting each year at the same place, at some central point, like the capital.

But there is another reason, which has not yet been alluded to, we believe, by any one. It is the want of money, on the part of the doctor, at the season of the year when the Association holds its meetings.

Texas is a pastoral State. The fields and flocks furnish the sum-total of export whence, only, money is brought into the State and turned loose, and hence this golden harvest comes, like Christmas, but once a year. It is well known that in the rural districts everything is done on a basis of "pay in the fall;" the doctors, with few exceptions, and these in the towns, where, there being railroad shops, or mills, or some other institution which disburses money monthly, medical men can collect more or less of their bills during the year, purchase on a credit everything they and their families consume! We know this to be true, and the almost universal rule is, to settle accounts in the fall, upon the sale of the crops or cattle; and, doctor bills being, notoriously, the last to be paid everywhere, are, in Texas, only paid in the fall—if ever.

Hence, it follows that "in the fall" is the only time when the country practitioner has money to spend, and to provide himself with books, instruments, etc. We know that the physicians of Texas are as liberal in the expenditure of money in the pursuit of their profession, and the acquisition of knowledge as any men on earth, and we believe that more of them would join the Association and attend the meetings if they had the money.

Why not, then, as our Constitution and By-Laws are now undergoing revision, and will come up at the approaching meeting, at San Antonio, for disposal, change the time of meeting from April to November? We really believe it would be a wise and judicious step; because, not only, as we have shown, do the physicians have more money to spare at the harvest time, when they get in their collections, but because the concensus of opinion, as gleaned from an extensive enquiry, goes to show that there is less sickness in the country along in the usually dry and bracing weeks of our glorious Indian summer than there is in the flowery and showery (and hot! oh, no!) month of April. We have observed closely, and made mental minutes of reasons given by doctors for not attending the meetings, and we honestly believe that the majority of members who fail to attend (when the meeting is near by) are detained at home by professional business; and we believe that it is *more than a conjecture*, that more accouchments take place in April than in November, and we all know how handy, and most frequently how true, the excuse is—"detained by an obstetric case."

We throw out the suggestion here, and hope members of the Association will consider it, and at the next meeting let it come up for discussion, pending the adoption of a new Constitution and By-Laws. It would be a departure from the cut and dried custom. Nearly all the State societies meet in April, and it would be interesting to know why. What advantage is there in meeting at that time? We believe we have shown that there would be an advantage in changing to the month of November. Let us try it, at least; for, if we ever hope to attract large meetings, or to enlist more than a decimal part of the practicing physicians of Texas in the cause, or to make stick those who join, we shall have to do something, and as the proposal to locate was rejected by the popular verdict, we now propose another plan. We believe the solution is to be found in the pocket-book: it is full in the fall, and empty in April.

---

THE NEW ORLEANS MEDICAL AND SURGICAL JOURNAL'S CRITICISM of the Louisiana State Medical Society, in the June number of that journal, is still rankling in the bosoms of some of the members ; and well it may. It was a cruel and unjust attack, inasmuch as it made no exceptions. The society's meetings were characterized as the "straggling together in some locality of a dozen or so languid, inconsequent, unprepared medical men, bent for the most part on a few days of rest, cigar smoking and story telling."

The subject is recalled by a circular letter to the profession by Dr. R. H. Day, in which he says, that he "joined some other members in writing to the editors, expressing condemnation of the editorial, feeling that it was unjust to those members who regularly attended the meetings and contributed to the best of their ability to make them attractive and instructive, hoping the editors would perceive their error and haste to retract its objectionable features," but that he "hoped in vain." Dr. Day then addresed the journal a letter for publication, and he states that the letter was never published, and that no notice was taken of his polite request for its return.

Under the circumstances this looks as if "insult has been added to injury"—and such treatment of a man of Dr. Day's age and professional standing, is a discourtesy of which we did not believe our talented contemporary could be guilty. Personally we know that Dr. Day is not an "inconsequent, unprepared medical man," and we believe that he, with many more members, have a higher purpose in attending the meetings than that attributed to them by our brilliant but in this instance, indiscreet, contemporary. Dr. Day held one of the highest offices in the late International Medical Congress; is a man seventy odd years of age; has practiced medicine over half a century, contributing voluminously to the medical literature of his time; was lately president of the Louisiana State Medical Society; organized, put in operation, and is president of the Physicians' Mutual Benefit Association of Louisiana; and taken all in all, no man stands higher in the medical profession, or has done more good to humanity and the cause of medicine than he. To refuse or fail to make a single exception even of such a man, in the bitter denunciation of the State Society by the only medical publication of the State, can be called by no other name than *rank injustice;* and we are surprised that the journal did not qualify its language, for it was evident that they had included in one sweep, every earnest, able and conscientious physician in the society, working for the interest of the profession. A little tact here might have saved much feeling, and perhaps many subscribers to the journal; amongst them men, who, like Dr. Day, have labored to uphold it and to make it useful and successful. If the journal had said "with a few brilliant exceptions," every member would have considered himself one of the exceptions.

---

WE TAKE OUR HAT OFF TO THE REGISTER.—The following is one of many complimentary notices of the Transactions, which have appeared in the Medical Press—and, by the bye,—is it not a little singular? We rarely ever see *such* notices of the Transaction of other States; why is it?

"TRANSACTIONS OF THE TEXAS STATE MEDICAL ASSOCIATION. Twentieth Annual Session, held at Galveston, Texas, April, 1888. Paper, 8 vo., pp. 300. Austin . Published for the Association.

"Although the transactions of the Society does not contain all the papers presented at the late meeting, yet there are a sufficient number of them to indicate that there is an activity which is commendable in this far-away section. A review of each paper would be impracticable, but it will suffice to say that the general character is of a high order, and they compare favorably with the work done by those in the more thickly-settled sections of the country. From a somewhat hasty perusal of some of them, we are inclined to believe that the advances in medicine and surgery and in the specialties find their way in Texas quite as readily as on the seaboard.

"The address of the President, Dr. Paine,* of Galveston, upon the subject, Medicine and Government, which he terms medical and political science—two branches of the main tree—is practical, scholarly, and eloquent, and our friends of the sunny South are to be congratulated upon having in their midst such an enthusiastic worker. The address in surgery and anatomy is in the nature of a review by Dr. Fly, of Galveston. There is, also,

---

*The Register should have said Dr. Sam R. Burroughs and not Dr. Paine; Dr. Paine is the President-elect, and will deliver the address at next meeting. We regret this error, as it does injustice to Dr. Burroughs.—ED.

a record of quite a number of important surgical operations performed in this section. The different sections in which papers were read (and we regret that the discussions have been omitted) are as follows: section of medical jurisprudence, psychology, and chemistry; a section on ophthalmology, and otology; one on practice, materia, medica, and theurapeutics; another on gynecology, on electro-therapeutics, and still another on dermatology. An appendix contains a roll of the members and a copy of the code of ethics, thus indicating to the world that they are still supporters of the American Medical Association and its principles."
—*Philadelphia Medical Register.*

---

MEDICAL LEGISLATION.—This subject will come up for disdussion at the next meeting of the Austin District Medical Society, December 20th. Dr. R. P. Talley, of Belton, Bell county, will read a paper on the subject, and the discussion will be opened by Dr. F. E. Daniel, by appointment. All the members will probably engage in the debate, and as there is a membership of over fifty, and a full attendance is expected, the discussion will doubtless take a wide range, and be full of interest. The representatives and senators elect of the district will be invited to be present, as well as several well known legal gentlemen of this and adjoining counties. Let every member come prepared to give his views as to the necessity or expediency of regulating the practice by statutory enactment.

---

DR. SWEARINGEN'S PAPER.—This admirable paper will be read with much interest and pleasure, not only by physicians, but by all who feel an interest in the subject which it so ably and fully presents. Copies of it will be sent to every member of the legislature, (which convenes in January). At the meeting of the Austin District Medical Society, October 20, upon which occasion the paper was presented, a committee was appointed to consider and report upon the best means of getting the subject fairly before the legislature, and of bringing about the accomplishment of the reform advocated. The committee consists of Drs. J.W. McLaughlin, (since elected president of the Society), W. A. Morris, and A. V. Doak, (of Williamson county).

